# RELIEF: A Digital Health Tool for the Remote Self-Reporting of Symptoms in Patients with Cancer to Address Palliative Care Needs and Minimize Emergency Department Visits

**DOI:** 10.3390/curroncol28060363

**Published:** 2021-10-21

**Authors:** Ravi Bhargava, Bonnie Keating, Sarina R. Isenberg, Saranjah Subramaniam, Pete Wegier, Martin Chasen

**Affiliations:** 1 Pharmaceutical & Healthcare Management and Innovation, Department of Biomedical and Molecular Sciences, Queen’s University, Kingston, ON K7L 3N6, Canada; ravi.bhargava@queensu.ca; 2 Global Institute of Psychosocial, Palliative and End-of-Life Care, University of Toronto, Toronto, ON M5S 1A4, Canada; 3 Division of Supportive and Palliative Care, William Osler Health System, Brampton, ON L6R 3J7, Canada; bonnie.keating@williamoslerhs.ca; 4 Bruyère Research Institute, Ottawa, ON K1N 5C8, Canada; sisenberg@bruyere.org; 5 Department of Medicine, University of Ottawa, Ottawa, ON K1H 8L6, Canada; 6 Institute of Health Policy, Management & Evaluation, University of Toronto, Toronto, ON M5S 1A4, Canada; pwegier@hrh.ca; 7 Department of Family & Community Medicine, University of Toronto, Toronto, ON M5S 1A4, Canada; 8 Humber River Hospital, Toronto, ON M3M 0B2, Canada; ssubramaniam@hrh.ca; 9 Department of Medicine, University of Toronto, Toronto, ON M5S 1A4, Canada; 10 Department of Family Medicine, McMaster University, Hamilton, ON L8S 4L7, Canada

**Keywords:** palliative care, home care, symptom reporting, remote monitoring, virtual care

## Abstract

The lack of timely symptom reporting remains a barrier to effective symptom management and comfort for patients with cancer-related palliative care needs. Poor symptom management at home can lead to unwanted outcomes, such as emergency department visits and death in hospital. We developed and evaluated RELIEF, a remote symptom self-reporting app for community patients with palliative care needs. A pilot feasibility study was conducted at a large, community hospital in Ontario, Canada. Patients self-reported their symptoms each morning using validated clinical symptom measures and RELIEF would alert for worsening or severe symptoms. RELIEF alerts were monitored by palliative care nurses who would then contact patients to determine if appropriate clinical intervention could be initiated to avoid unnecessary emergency department visits. A total of 20 patients were recruited to use RELIEF for two months. Patients completed 80% of daily self-report assessments; 133 alerts were trigged, half of which required clinical intervention. No patient visited the emergency department for symptom management during the study. Clinical staff estimated five emergency department visits were avoided because of RELIEF—saving an estimated cost of over CAD 60,000. RELIEF is a feasible and acceptable method for the remote monitoring of patients with palliative care needs through regular symptom self-reporting.

## 1. Introduction

Palliative care represents a key aspect for the delivery of people-centred healthcare and has been shown to improve quality of life and prevents and relieves suffering through the early identification, assessment, and treatment of physical, psychosocial, or spiritual symptoms [1]. The effective delivery of palliative care requires patient-centred communication, whether it be with palliative care physicians and nurses, oncologists, or social workers [2,3]. Over 90% of Canadians have agreed patients have the right to receive care in their own homes at the end of life and over half of Canadians expect the bulk of their end-of-life care will occur in their own homes [4]. To achieve this goal of home-based care, patients require frequent assessments—assessments which provide more timely and earlier interventions for patients by their clinician should an intervention be required. However, the primary challenge is the requirement for sufficient resources and services around symptom assessment, monitoring, and management. 

A key barrier to effective symptom management and patient/family comfort is the lack of real-time symptom status. Most patients must visit the physician or other care provider either in the clinic or have the provider come to the home for assessments. Limited assessments may lead to more frequent emergency visits by patients/caregivers, while more frequent assessments would provide more timely and earlier interventions for patients by their clinician should an intervention be required [5]. Poor symptom monitoring in the home and late referrals for symptom management may account for many patients dying in hospital—nearly 65% of patients in Ontario died in hospital per one study, with substantially higher costs to the healthcare system in these hospitalized patients [5].

Research shows that remote monitoring of symptoms can improve care in patients with palliative care needs. Electronic forms of capturing symptoms have been associated with improvements in cancer symptom outcomes. A recent study found significant improvement in self-reported quality of life, reduced visits to the emergency department, and increased survival among chemotherapy patients who received weekly emails for symptom monitoring, compared to those receiving usual care [6]. Another compared survival in patients with a history of lung cancer who were followed by a web-based algorithm for the early detection of relapse and early palliative care initiation—based on weekly self-reported patient symptoms—and found survival was significantly better in this intervention group than in the control group with an overall median survival of greater than five months [7].

We developed the Remote Self-Reporting of Symptoms by Patients with Palliative Care Needs (RELIEF) app as an innovative e-health solution to address issues around routine patient symptom assessment in patients with palliative needs. We had two goals in this pilot work: (1) to demonstrate RELIEF to be a feasible tool for patients with palliative care needs to easily self-report their symptoms to their clinical team; and (2) to allow for timely interventions or close monitoring by clinical staff to minimize unnecessary visits to the emergency department or admissions to hospital in this patient population.

## 2. Methods

### 2.1. Design

RELIEF is a web-based application for symptom self-reporting by patients with palliative care needs. We developed RELIEF using human-centred design processes; without a focus on any one symptom, disease, or disease stage; and with a focus on seamless integration into the clinical workflow for healthcare providers. The patient or their caregiver securely logs into the site every day to self-report symptoms, distress, and pain using a set of validated clinical measures. That data are reported to the patient’s healthcare providers and increases in symptom burden, distress, or pain are flagged for clinical review. Providers receive RELIEF alerts for any sudden changes in patient status, or if the patient’s symptoms, distress, or pain have been trending in a negative direction. We hoped that with these frequent assessments through RELIEF, it would allow for: (1) earlier intervention; (2) mobilization of auxiliary services; and (3) recommendation for emergency or palliative intake. 

### 2.2. Measures

Once a patient or their caregiver logged into RELIEF, they completed three self-reported symptom scales. All results were then made available for the patient’s clinical team to review.

#### 2.2.1. Edmonton Symptom Assessment System

The Edmonton Symptom Assessment System Revised (ESAS-r) [8] is a 10-item self-report measure for common patient symptoms, such as pain, drowsiness, and shortness of breath. An additional Other category exists to capture any other symptom (e.g., constipation). Items are rated using an 11-point visual analogue scale, with values ranging from 0 (not at all) to 10 (worst possible).

#### 2.2.2. Distress Thermometer

The Distress Thermometer (DT) [9] is a self-report visual analogue scale resembling a thermometer, with values ranging from 0 (no distress) to 10 (extreme distress). A score of 4 on the DT was found to offer the best sensitivity and specificity for identifying potentially high distress [10].

#### 2.2.3. Brief Pain Inventory

The Brief Pain Inventory (BPI) [11] is a questionnaire regarding patient pain levels and their effect on function, allowing for the assessment of the interference of pain on patient affect in addition to patient activities.

### 2.3. Setting

We conducted the study at the Brampton Civic Hospital in Brampton, Ontario, Canada, which is part of the William Osler Health System, a network of hospitals within Ontario. This health system serves a diverse population of more than 2 million residents—nearly 40% are 65 years old or older, the minority populations include Southeast Asian, Black, Latin American, and Filipino; and the median after-tax household income is less than CAD 60,000.

### 2.4. Ethical Considerations

This study underwent a formal review and approval process by the Research Ethics Board of the William Osler Health System (#18-0028). All participants provided a written signed informed consent and verbally confirmed at the start of the study.

### 2.5. Procedure

#### 2.5.1. Recruitment of Patients

Eligibility criteria included those who were: (1) aged 18 years or older; (2) alert and oriented; (3) able to understand and communicate in English or have a family member who could; and (4) able to access a desktop, laptop, smartphone, or tablet with internet access. Patients who had moderate to severe confusion (acute as in a delirium or chronic as in a dementia) that would interfere with the ability to follow instructions were excluded from participating in this pilot study, as they may have been unable to comprehend and retain the overall purpose of the study, the directions to complete the ESAS, DT, and BPI, and the ability to communicate with his/her health care professionals. All patients who attended the Brampton Civic Hospital Palliative Care Clinic or were newly referred to the clinic, and who met eligibility criteria, were approached in person by a clinical nurse specialist (B.K.) after their appointment. Patients who expressed an interest in participating in the study were given an information sheet describing the study and the roles of the patient, nurse, and physician. Information sheets were reviewed with the patient.

#### 2.5.2. Onboarding and Training

If a patient provided written consent to participate in the study, they were provided with training on how to use RELIEF. Palliative clinical nurses demonstrated how RELIEF worked and trained patients how to use it to complete the ESAS-r, DT, and BPI. A teach back approach was used to ensure the patient knew how to properly use RELIEF prior to leaving the clinic. An instructional booklet about this training was also provided to patients and their caregivers. Patients were instructed to complete their assessments daily at a time that was convenient for them and their family, but preferably between the hours of 6 AM and 8 PM. Patients were provided with contact information if they required support, along with the contact information for the clinic and after-hours service.

#### 2.5.3. Monitoring

RELIEF would flag a patient for review and alert their healthcare provider if the patient reported (1) an increase of 2 points each day over 2 consecutive days; (2) an increase of 3 points over the previous day; or (3) any score of 8 or higher, for any of the symptoms listed in the ESAS-r, DT, or BPI. RELIEF was monitored from 8 AM to 5 PM, Monday to Friday, by palliative care nurses who were part of the study. Nurses responded to RELIEF alerts with a telephone call to the patient or family and completed an over-the-phone assessment of the patient. This assessment verified the reasons for the alert and included an in-depth symptom assessment—including quantitative and qualitative characteristics—and a focused review of current medications for the symptom which triggered the alert. A review of the patient’s goals of care was also carried out, followed by a discussion with the patient and family to discuss the plan to address the symptoms. The palliative care nurses offered pain and symptom management within their scope of practice; however, if physician intervention was required, the attending palliative care specialist was contacted. Any initiated interventions were documented in the patient’s electronic medical record. For alerts triggered after hours or on weekends, the palliative care physician team initiated the same assessment process described above. For the purposes of this pilot study, patients recruited into the study were able to use RELIEF for a two-month follow-up period and completed the self-report symptom assessment daily on RELIEF.

### 2.6. Analysis

All analyses were conducted using R [12].

## 3. Results

### 3.1. Participants

A total of 42 patients were screened. The reasons for non-recruitment included: language barriers; lack of smart device or lack of knowledge to use smart device; no internet access; lack of family support at home to assist with completing forms; too many medical appointments or too busy; too “taxing” for family; afraid they would forget to do daily reporting; too fatigued; overwhelmed at first clinic visit; not interested; or too high functioning or no/low symptom burden.

We recruited 20 patients from the Supportive Palliative Care Clinic at Brampton Civic Hospital; however, four patients never used the RELIEF app, and three patients withdrew from the study. Characteristics for the final sample of 13 patients are shown in Table 1. While RELIEF was designed as a disease-agnostic app, all patients had a cancer diagnosis, as this represented the bulk of the patients in the Supportive Palliative Care Clinic at Brampton Civic Hospital.

### 3.2. Use and Usability of RELIEF

We found that across the two-month follow-up period for this pilot study, 80% of daily symptom self-report assessments were completed. The 20% non-adherence rate was related to factors unrelated to RELIEF itself but rather with the health of the patient. Patients who were high functioning and with low symptom burden were most likely to withdraw from the study and/or not complete the forms daily. Three patients withdrew because of no/low symptom burden and because they found this exercise very repetitive. See screenshot of a patient completing RELIEF assessments in Figure S1.

Patients low in function and/or those experiencing significant fatigue were also likely to withdraw from the study and not complete their forms daily. The most common reasons reported for not completing forms were, “too fatigued”, “forgot to”, “other priorities at this time in my life”, and “too many medical appointments to attend”. Four patients never used the application because of either disease progression, or they died only a few days after enrolment in this study.

We surveyed the palliative care nurses and physicians who participated in the pilot about the experience using RELIEF—92% of clinicians reported improved confidence in providing care and an improved client experience and 75% of clinicians perceived improved quality of life for their patients. There were also four calls made by patients and clinical staff for technical support.

### 3.3. RELIEF Alerts and Intervention

Over the two-month follow-up period, a total of 133 alerts were triggered for the patients on RELIEF. Of those, 66 (49.6%) required clinical intervention: 60 resulted in over-the-telephone assessments and interventions; 5 resulted in urgent clinic visits for pain and symptom management; 1 resulted in an urgent home visit by a community nurse. Two patients were provided support for expected death in the home. Healthcare provider dashboard, showing which patients have RELIEF alerts was shown in Figure S2.

The remaining 67 alerts did not require intervention. The palliative care nurses involved in the study used RELIEF to review the past five days of symptom scores for the patients and then accessed the electronic medical record of the patient for treatment plans to determine whether intervention was appropriate. Nurses also had access to the clinic schedule and thus had knowledge that patients for whom RELIEF alerted would be in the clinic later that day and would receive appropriate medical attention.

In addition to the alerts provided by RELIEF, we observed instances of clinical intervention being initiated without an alert: seven telephone calls were initiated by palliative care nurses because of their monitoring RELIEF and noticing worrying results, and there were six instances of patients calling the palliative care nurses involved in the study for symptom support.

One patient went to the emergency department for reasons unrelated to symptom management (subcutaneous site restart for a PCP pump). Across the two-month follow-up period, four direct admissions from home were made for study patients, two patients died while at home, and one patient died after being admitted to the hospital. None of the patients visited the emergency department for reasons related to symptom management during the study.

### 3.4. Cost Avoidance

Five patients in the study experienced highly active symptoms over several days. These patients required frequent clinical support and interventions by various palliative care team members. Those clinicians agreed that with RELIEF, each patient was able to be managed in the home, and not only were emergency department visits prevented, but at least one admission to hospital was avoided in each case. With the help of the Decision Support and Finance departments at Brampton Civic Hospital, where this study was based, we estimated the healthcare cost avoidance due to the patients being monitored through RELIEF. The full cost of a palliative admission via the emergency department at our study site averaged CAD 850.00 per day, with an average length of stay of 14.61 days, with five patients experiencing high symptom burden, resulting in savings of CAD 62,092.50. All reported amounts are in Canadian dollars. Details of what symptoms triggered the RELIEF alert was shown in Figure S3.

## 4. Discussion

RELIEF was built with the goal to enable the remote self-reporting of symptoms for patients with palliative care needs, without a focus on any one symptom, disease, or disease stage, and with a focus on seamless integration into the clinical workflow for healthcare providers. Our pilot work here demonstrated that (1) RELIEF is a feasible tool for the remote self-reporting of symptoms by patients with palliative care needs; and (2) Timely clinical interventions can be initiated by monitoring patients through RELIEF and the alerts generated by RELIEF.

We showed RELIEF is a highly useable tool for both patients and clinicians. We found that 80% of symptom assessments were completed by patients, meaning patients found RELIEF to be an acceptable method to routinely report their symptoms in this way. RELIEF was similarly acceptable to clinicians—92% reported improved confidence in providing care and an improved client experience and 75% perceived improved quality of life for their patients. RELIEF was also easy to use, as only four calls for technical support were made by both patients and clinical staff. 

The current standard of care includes in-person clinic appointments scheduled an average of 4 weeks apart and based on patient need at the time of the in-clinic visit. Most patients have had community support via a home-based palliative homecare program. Community palliative nurses contact the palliative physician when needed. As patients become home bound, their care transitions from clinic appointments to palliative physician home visits.

RELIEF allowed for the timely initiation of appropriate clinical interventions to occur. Half of the 133 alerts triggered by RELIEF resulted in a clinical intervention being initiated. For the remainder, palliative care nurses were able to determine that clinical intervention was not needed or that the patient would be seen the same day by clinic staff and thus receive the appropriate medical care. Moreover, the option to monitor the dashboard at all times allowed for clinicians to determine when telephone follow-up was required outside of a triggered alert. Finally, RELIEF allowed several emergency department visits and hospital admissions to be avoided, resulting in significant cost avoidance to the healthcare system.

Despite the feasibility and acceptability of RELIEF, 3 of the 20 patients initial recruited withdrew from the study. These patients were either too sick to use RELIEF or too high functioning to see the need for it. Thus, we believe the ideal population to be one with a palliative performance scale [13,14] score of 30–70%.

The COVID-19 pandemic exposed many difficulties of managing the care of patients with palliative needs, especially when the patients do not require hospitalization. Studies have found that remote patient monitoring solutions are highly feasible and well received by patients [15]; more than 90% of Canadians are willing to use virtual care as an alternative to in-person visits [16], and Canadians appear to be highly satisfied with virtual care and up to one-third would like virtual care to be the first point of contact [17].

RELIEF could improve equity in the Canadian healthcare system as patients who may otherwise be underserved locally are able to access high-quality palliative care support via remote monitoring and timely acute interventions. Patients may no longer be limited in their ability to access high-quality palliative care services because of their geographic location, socioeconomic status, or healthcare needs. Healthcare provider capacity will also increase, as the same provider will be able to monitor and address the issues of a larger number of patients due to the workflow improvements RELIEF provides.

### Limitations & Future Directions

First, our patient population was primarily an English-speaking oncology population and the patients were recruited from a large, well-resourced, urban hospital. Additional work is being undertaken to demonstrate the replicability of our success here across diverse populations and settings, including the homeless and marginally housed; rural and remote communities; Indigenous communities; non-English speaking patients; and non-cancer patients. RELIEF will provide real-time communication of patient symptoms to clinical staff for proactive prevention, monitoring, and intervention, while reducing patient stress in knowing their healthcare provider is monitoring their symptoms. 

## 5. Conclusions

We demonstrated that RELIEF is a feasible and acceptable tool for the remote self-reporting of symptoms by patients with palliative care needs. RELIEF allowed for symptom assessments to be easily collected from patients, troubling scores to be flagged for clinical review, and the appropriate intervention to be initiated in a timely manner. Patients were able to be monitored from home and we showed significant cost avoidance to the healthcare system as a result. RELIEF has the potential to improve equity in the delivery of healthcare—the patients who need the attention receive it as needed while the patients who are doing well can be monitored for any potential declines.

## Figures and Tables

**Table 1:** Characteristics of RELIEF patients.

Characteristic	
*n*	13
Age—Mean (SD)	63 (9)
Age range	52–74
Sex—% (*n*)	
Female	46% (6)
Male	54% (7)
Survival to study completion—% (*n*)	85% (11)
Diagnoses (*n*)	
Metastatic lung cancer	2
Metastatic breast cancer	4
Metastatic pancreatic cancer	3
Metastatic ovarian cancer	1
Metastatic rectal cancer	1
Multiple myeloma	1
Metastatic cholangiocarcinoma	1

## Data Availability

No data are available.

## Supplementary Materials

The following are available online at https://www.mdpi.com/article/10.3390/curroncol28060363/s1, Figure S1: Screenshot of a patient completing RELIEF assessments, Figure S2: Healthcare provider dashboard, showing which patients have RELIEF alerts, Figure S3: Details of what symptoms triggered the RELIEF alert.
