# Supplementary material for: RELIEF: A Digital Health Tool for the Remote Self-Reporting of Symptoms in Patients with Cancer to Address Palliative Care Needs and Minimize Emergency Department Visits
Source: Curr Oncol. 2021 Oct 21;28(6):4273–80. doi: 10.3390/curroncol28060363 (PMC8544531; doi:10.3390/curroncol28060363)
Supplement: Supplementary file 1 [file curroncol-28-00363-s001.zip › curroncol-1364976-supplementary.pdf]

Current Oncology

Supplementary Material

RELIEF: A Digital Health Tool for the Remote Self-Reporting of Symptoms in Patients with Cancer to Address Palliative Care Needs and Minimize Emergency Department Visits

Ravi Bhargava, Bonnie Keating, Sarina R. Isenberg, Saranjah Subramaniam, Pete Wegier and Martin Chasen

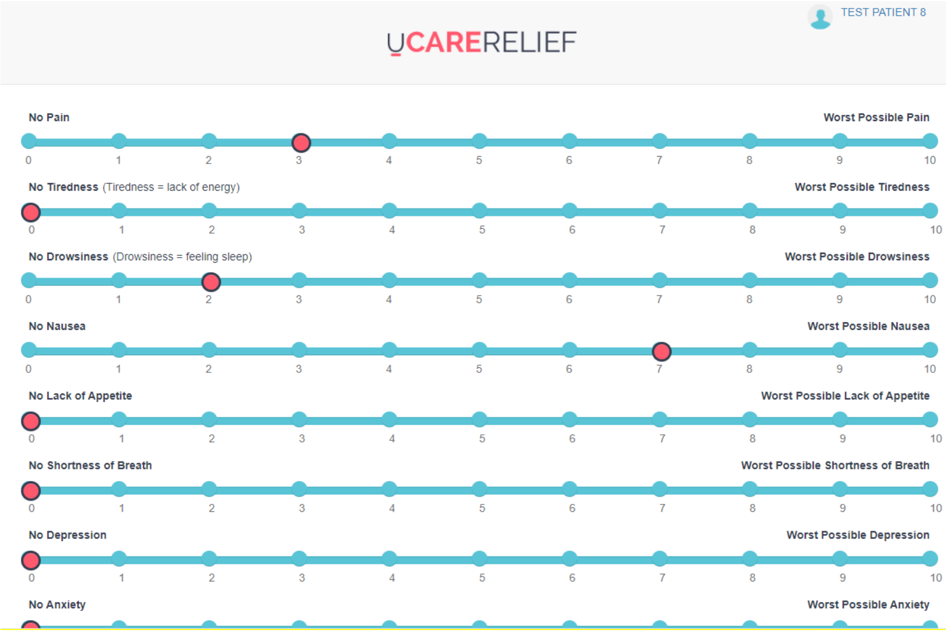

Figure S1. Screenshot of a patient completing RELIEF assessments.

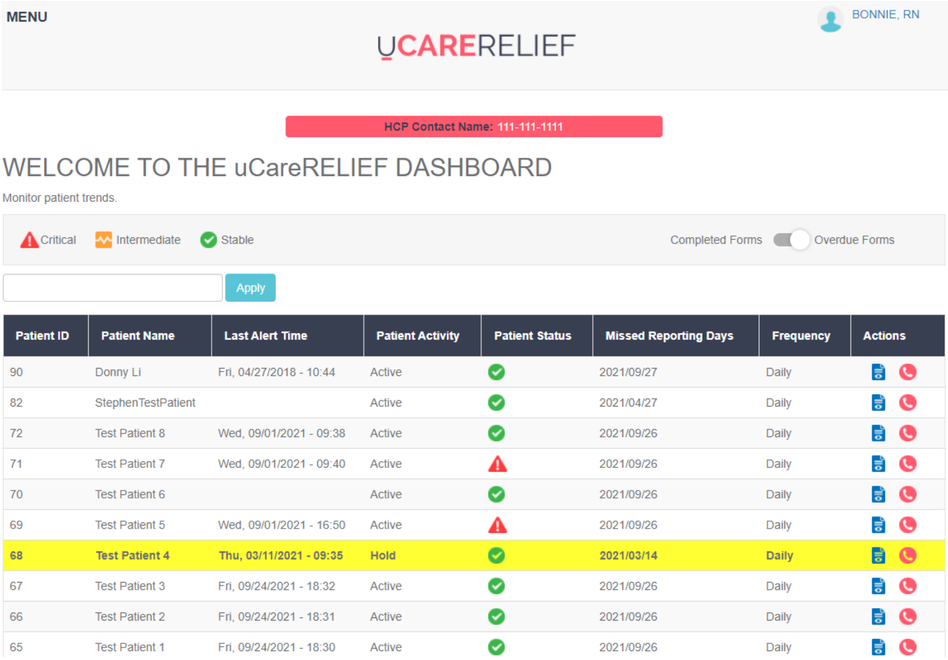

Figure S2. Healthcare provider dashboard, showing which patients have RELIEF alerts.

Copyright: © 2021 by the authors. Licensee MDPI, Basel, Switzerland. This article is an open access article distributed under the terms and conditions of the Creative Commons Attribution (CC BY) license (https://creativecommons.org/licenses/by/4.0/).

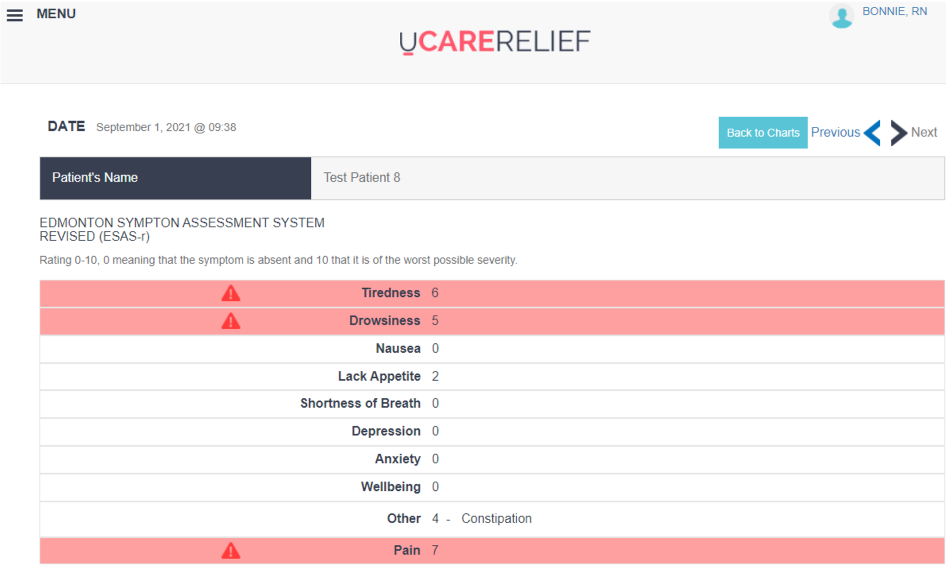

Figure S3. Details of what symptoms triggered the RELIEF alert.
